# High mobility group box 1 and a network of other biomolecules influence fatigue in patients with Crohn’s disease

**DOI:** 10.1186/s10020-023-00679-6

**Published:** 2023-06-26

**Authors:** Ingeborg Kvivik, Tore Grimstad, Kjetil Bårdsen, Grete Jonsson, Jan Terje Kvaløy, Roald Omdal

**Affiliations:** 1 Research Department, Stavanger University Hospital, P.O. Box 8100, 4068 Stavanger, Norway; 2 Unit of Gastroenterology, Department of Internal Medicine, Stavanger University Hospital, Stavanger, Norway; 3 Department of Clinical Science, Faculty of Medicine, University of Bergen, Bergen, Norway; 4 Department of Medical Biochemistry, Stavanger University Hospital, Stavanger, Norway; 5 Department of Mathematics and Physics, University of Stavanger, Stavanger, Norway; 6 Department of Rheumatology, Stavanger University Hospital, Stavanger, Norway

**Keywords:** HMGB1, Chronic inflammation, Principal component analysis, Crohn’s disease, Fatigue, Sickness behavior, Cellular defense

## Abstract

**Background:**

Fatigue is common in patients with chronic inflammatory and autoimmune diseases, often with a severe impact on the patient’s daily life. From a biological point of view, fatigue can be regarded as an element of the sickness behavior response, a coordinated set of responses induced by pathogens to enhance survival during an infection and immunological danger. The mechanisms are not fully understood but involve activation of the innate immune system, with pro-inflammatory cytokines, in particular interleukin (IL)-1β, acting on cerebral neurons. These mechanisms are also active during chronic inflammatory conditions. High mobility group box 1 (HMGB1) protein has interleukin-1 like properties and is a strong inducer of innate immune responses. Its role in generation of fatigue is not clarified. Emerging evidence indicates that also other biomolecules may influence sickness behavior. We aimed to elucidate how HMGB1 influences fatigue in patients with Crohn’s disease, and how the protein interacts with other candidate biomarkers of fatigue.

**Methods:**

In 56 patients with newly diagnosed Crohn’s disease, fatigue was evaluated using three different fatigue instruments: the fatigue visual analog scale (fVAS), Fatigue Severity Scale (FSS), and the vitality subscale of Medical Outcomes Study Short-Form Health Survey (SF-36vs). The biochemical markers IL-1 receptor antagonist (RA), soluble IL-1 receptor type 2 (sIL-RII), heat shock protein 90 alpha (HSP90α), HMGB1, anti-fully reduced (fr)HMGB1 antibodies (abs), hemopexin (HPX), and pigment epithelium-derived factor (PEDF) were measured in plasma. Multivariable regression and principal component analyses (PCA) were applied.

**Results:**

Multivariable regression analyses revealed significant contributions to fatigue severity for HMGB1 in the FSS model, HSP90α in the fVAS model and IL-1RA in the SF-36vs model. Depression and pain scores contributed to all three models. In PCA, two components described 53.3% of the variation. The “inflammation and cellular stress dimension” was dominated by IL-1RA, sIL-1RII, HSP90α, HPX, and PEDF scores, where the “HMGB1 dimension” was dominated by HMGB1, anti-frHMGB1 abs, and fVAS scores.

**Conclusion:**

This study supports the hypothesis that HMGB1 and a network of other biomolecules influence fatigue severity in chronic inflammatory conditions. The well-known association with depression and pain is also acknowledged.

**Supplementary Information:**

The online version contains supplementary material available at 10.1186/s10020-023-00679-6.

## Background

Fatigue, a phenomenon characterized by abnormal tiredness, lack of energy, and feeling of exhaustion, is common in cancer, neurodegenerative, chronic inflammatory, and autoimmune diseases (Krupp and Pollina 1996; Overman et al. 2016). From a biological point of view, fatigue can be considered as part of the “sickness behavior response”, a phenomenon characterized by fatigue, weariness, depression, lack of thirst and hunger, and social withdrawal that occurs in animals and humans during infection and bodily damage. These adaptive responses are beneficial for survival and have been under intense selective genetic pressure during evolution (Hart 1988; Medzhitov and Janeway 1997). In acute conditions, this behavior is beneficial but becomes purposeless in chronic diseases in which the same mechanisms that drive sickness behavior are active.

Both animal and human studies have demonstrated the important role of pro-inflammatory cytokines in fatigue, particularly interleukin (IL)-1β (Heesen et al. 2006; Dantzer et al. 2014; Roerink et al. 2017). When this molecule passes from the blood to brain or is released by microglia in the brain, it will bind to brain-specific IL-1 receptors on neurons and induce sickness behavior and fatigue (Smith et al. 2009). In addition to IL-1β itself, other molecules like IL-1 receptor antagonist (RA) and soluble IL-1 receptor type 2 (sIL-RII), modulate IL-1 activities and influence sickness behavior (Bårdsen et al. 2019).

The high mobility group box 1 (HMGB1) protein can also be a strong inducer of IL-1β. HMGB1 is an alarmin/damage associated molecular pattern (DAMP) protein that is released from damaged cells and activated immune cells (Wang et al. 1999; Andersson et al. 2018; Gardella et al. 2002). The redox-status of HMGB1 is crucial for its functions, the disulfide (ds)HMGB1 is the immune active variant, and the fully reduced (fr)HMGB1 works as a chemoattractant (Yang et al. 2010; Schiraldi et al. 2012). Both variants are strong activators of the immune system by binding receptors like Toll-like receptor (TLR)4 and receptors for advanced glycation end products (RAGE) on the cell surface of immune cells. This binding will lead to down-stream production and release of IL-1β, tumor necrosis factor (TNF)-α and IL-6, all classical proinflammatory cytokines, it is therefore likely that HMGB1 also participates in the fatigue inducing processes (Ghosh et al. 2022; Yang et al. 2013; He et al. 2018). A mechanism for regulating HMGB1 is production of autoantibodies against HMGB1. We have previously shown that high levels of anti-HMGB1 antibodies (abs) in plasma from patients with Crohn’s disease are negatively associated with high fatigue (Kvivik et al. 2021), suggesting a protective/down-regulating role for these specific abs.

Molecules that are not directly pro-inflammatory also influence fatigue. An example is heat shock protein 90 alpha (HSP90α), a ubiquitous molecular chaperone involved in protein folding and downregulation of cellular stress and immune responses (Grimstad et al. 2020). Other examples are two molecules involved in cellular stress responses, namely pigment epithelium-derived factor (PEDF) and hemopexin (HPX), which have been found in cerebrospinal fluid (CSF) and are associated with fatigue (Larssen et al. 2019). Such observations suggest that anti-inflammatory mechanisms and cellular stress responses also influence fatigue. Conceivably, rather than one single and major “fatigue-inducing” molecule, several biomolecules may work together in a fatigue-regulating network. To test this hypothesis, we measured select candidate plasma biomarkers and used multivariable statistics to explore fatigue in patients with Crohn’s disease, a chronic inflammatory disease that may affect any part of the gastrointestinal tract (Baumgart and Sandborn 2007). Fatigue is among the most frequent and bothersome complaints in this disease (Romberg-Camps et al. 2010; Grimstad et al. 2015).

## Methods

### Patients and study design

Fifty-six patients with newly diagnosed Crohn’s disease at the Unit of Gastroenterology, Stavanger University Hospital, Norway, were consecutively included in this study from January 1, 2013, to December 31, 2016. Inclusion criteria were age ≥ 16 years and newly diagnosed Crohn’s disease based on a combination of clinical, biochemical, stool, endoscopic, radiological, and histological findings suggestive of Crohn’s disease (Maaser et al. 2019). Four patients had autoimmune co-morbidity; two with ankylosing spondylitis, one with systemic lupus erythematosus and one with psoriasis. No patients were on corticosteroids, immune modulators (azathioprine or methotrexate) or biological drugs. The exclusion criteria were inability to adhere to the study protocol, previous history of inflammatory bowel disease, and pregnancy. After inclusion, the participants underwent a single study visit with colonoscopy, and clinical, laboratory, and endoscopic data were recorded. All study data were recorded within 3 days after the visit.

### Fatigue, pain, and depression assessments

Fatigue was rated by the fatigue visual analog scale (fVAS), the Fatigue Severity Scale (FSS), and the vitality subscale of Medical Outcomes Study Short-Form Health Survey (SF-36) (Wolfe 2004; Hewlett et al. 2011). The fVAS is a 100-mm horizontal line with vertical endpoints and the wording “No fatigue” on the left end and “Fatigue as bad as it can be” on the right end. The patients are asked to draw a vertical line at the point that corresponds with the level of fatigue they experienced the previous week. The fVAS score is the distance from the left end to the drawn line in millimeters. FSS is a questionnaire in which the patient is asked to respond to nine items, each with a score of 1–7. The mean score of the nine items is the FSS score. Higher FSS scores indicate more fatigue. The vitality subscale of SF-36 (SF-36vs) addresses the subjects’ energy and feeling of being worn out in the last month, whereas the SF-36 subscale for bodily pain (SF-36bp) rates the pain severity (Wolfe 2004). Both subscales range from 0 to 100, with higher scores corresponding to less fatigue or less pain, respectively. The SF-36 subscale scores were inverted to be more coherent with the other measurements. Mood was evaluated by the depression subscale of the Hospital Anxiety and Depression scale (HADS-D) in which seven items are rated from 0 to 3, and a sum score ≥ 8 indicates clinical depression (Bjelland et al. 2002).

### Inflammatory biomarkers

Inflammatory activity was assessed by C-reactive protein (CRP) in blood ± 3 days from the study visit and fecal (f)-calprotectin within a time frame of 4 weeks before and 3 days after the study visit.

### Disease activity assessment

The Harvey Bradshaw Index (HBI) was recorded for all study participants. This symptom-based index includes the following items: general well-being, abdominal pain, number of loose stools the previous day, the presence of a palpable mass in the abdomen, and extra-intestinal manifestations (arthralgia, fistula, and abscess) (Harvey and Bradshaw 1980).

The Simple Endoscopic Score for Crohn’s Disease (SES-CD) was also recorded to evaluate the grade of intestinal inflammation as an objective rating of disease activity (Daperno et al. 2004).

### Blood sampling and biochemical analyses

EDTA-blood was centrifuged at 2800 g for 15 min at 4 °C and plasma samples stored immediately at − 80 °C in aliquots until further analysis. Based on previous experiences and findings in our laboratory regarding potential biomarkers for fatigue (Bårdsen et al. 2019, 2016; Kvivik et al. 2021; Grimstad et al. 2020; Larssen et al. 2019; Harboe et al. 2009) we selected a panel of relevant candidate proteins. Plasma concentrations of IL-1RA were measured by a sandwich immunoassay with electrochemiluminescence detection on a Meso^®^ QuickPlex SQ 120 Imager (Meso Scale Discovery, Rockville, MD). Antibodies in plasma against frHMGB1 were measured by an in-house ELISA method (Kvivik et al. 2021). Briefly, 96-well plates were coated with recombinant frHMGB1 (HMGBiotech, Milan, Italy) to capture anti-frHMGB1 abs. The absorbance of developed color after adding o-phenyldiamine and peroxidase to each well was used as a semi-quantitative measure of anti-frHMGB1 abs in plasma. sIL-1RII, HSP90α, HMGB1, PEDF, and HPX were analyzed using commercially available ELISA kits (sIL-1RII: R&D Systems, Minneapolis, MN; HSP90α: Enzo Life Sciences, Farmingdale, NY; HMGB1: IBL International, Hamburg, Germany; PEDF and HPX: Nordic Biosite, Täby, Sweden). The reproducibility of the ELISA methods was monitored by analyzing two plasma controls in triplicate on each plate. The inter-assay coefficients of variation (CV) for sIL-1RII, HSP90α, HMGB1, PEDF, and HPX were < 12%, and for anti-frHMGB1 abs < 17.8%. Plasma was diluted 1:25, 1:500, and 1:50 prior to analysis of sIL-1RII, PEDF, and HPX, respectively. Otherwise, the samples were handled as recommended by the manufacturer. The absorbance was measured at the recommended wavelengths by Biotek Synergy H1 (Agilent, Santa Clara, CA). Results below the limit of detection (LOD) for anti-frHMGB1 abs and HMGB1 were imputed as LOD/√2 to enable calculations.

### Statistical analysis

The normal distribution of data was tested using the Shapiro–Wilk test. Normally distributed variables are presented as mean and standard deviation (SD), other data as median and range. Associations between clinical measures, biochemical variables, and fatigue were first explored using age, SES-CD, HBI, CRP, f-calprotectin, SF-36bp, HADS-D, IL-1RA, sIL-RII, HSP90α, HMGB1, anti-frHMGB1 abs, PEDF, and HPX as independent variables, and the fatigue measures (fVAS, FSS, and SF-36vs scores) as dependent variables in univariable linear regression analyses. Independent variables with p-values < 0.1 in the univariable analyses were then included in three separate multivariable regression models using fVAS, FSS, and SF-36vs scores, respectively as dependent variables. Finally, forward and backward selection models were used to suggest models with only significant independent variables. Goodness of fit was verified by the analysis of residuals.

Principal component analysis (PCA) was performed to increase the interpretability and retain potential trends and patterns of complex associations between the biochemical variables and fatigue scores. Missing values were imputed using simple imputation, and Hotelling’s T-test for outliers was performed. Statistical analyses were performed using SPSS version 28.0.1.0 and R version 4.1.2. The R packages missMDA, FactoMineR, Factoshiny, and ropls were used for imputation and PCA.

## Results

Clinical characteristics and laboratory data are given in Table 1.

**Table 1:** Demographic, clinical, and biochemical variables in 56 patients with newly diagnosed and untreated Crohn’s disease

Variable	
Age, years	33.5 [16–78]
Male/female	24 (42.9%)/32 (57.1%)
SES-CD	7 [1–37]
HBI (n = 55)	5 [0–14]
Disease distribution	
Ileum	29 (51.8%)
Colon	6 (10.7%)
Ileocolon	21 (37.5%)
CRP, mg/L	8.5 [1–139]
F-calprotectin, mg/kg (n = 52)	278 [15–4432]
fVAS score	50.5 (25.6)
FSS score	4.0 (1.8)
SF-36vs score^a^	61.4 (22.9)
SF-36bp score^a^	59 [0–100]
HADS-D (n = 55)	3 [0–13]
IL-1RA, pg/mL	292.2 [126.2–1412.3]
sIL-1RII, ng/mL	6.78 [3.29–49.90]
HSP90α, ng/mL (n = 52)	17.28 [6.44–55.06]
HMGB1, ng/mL (n = 52)	1.17 [0.22–3.33]
Anti-frHMGB1 abs^b^ (n = 49)	0.040 [0.040–1.179]
PEDF, ng/mL (n = 46)	655 [458–1100]
HPX, ng/mL (n = 46)	735.3 (224.5)

Values are given as n (%), median [range], or mean (SD)

^a^SF-36vs- and SF-36bp scores are reported as inverted values, with high numbers indicating low vitality/high bodily pain and low numbers indicating high vitality/low bodily pain. ^b^Absorbance at 490 nm

*abs* antibodies, *f-calprotectin* fecal calprotectin, *frHMGB1* fully reduced HMGB1, *fVAS* fatigue visual analog scale, *FSS* Fatigue Severity Scale, *HADS-D* depression subscale of the Hospital Anxiety and Depression Scale, *HBI* Harvey Bradshaw Index, *HMGB1* high mobility group box 1, *HPX* hemopexin, *HSP* heat shock protein, *IL-1RA* interleukin-1 receptor antagonist, *sIL-1RII* soluble interleukin-1 receptor type 2, *PEDF* pigment epithelium-derived factor, *SES-CD* Simple Endoscopic Score for Crohn’s Disease, *SF-36* Medical Outcomes Study Short-form Health Survey, *SF-36bp* the bodily pain subscale of SF-36, *SF-36vs* the vitality subscale of SF-36

In results from all three fatigue instruments, univariable linear regression analyses revealed positive associations between fatigue scores and HBI, HADS-D, and SF-36bp scores, and negative associations with anti-frHMGB1 ab concentrations (Table 2). HSP90α concentrations were positively associated with fVAS scores and HMGB1 concentrations with FSS scores. In addition, SES-CD scores, CRP, f-calprotectin, IL-1RA, and HPX concentrations were positively associated with SF-36vs scores.

**Table 2:** Associations between fatigue scores and clinical and biochemical variables in 56 patients with Crohn’s disease

	Dependent variables								
	fVAS			FSS			SF-36vs^a^		
	B	R^2^	p-value	B	R^2^	p-value	B	R^2^	p-value
Age, years	− 0.37	0.05	0.11	− 0.02	0.02	0.36	− 0.02	0	0.94
SES-CD	0.56	0.03	0.20	0.06	0.06	0.07	**1.21**	**0.17**	**0.001**
HBI (n = 55)	**2.04**	**0.09**	**0.02**	**0.19**	**0.16**	**0.002**	**2.31**	**0.15**	**0.004**
CRP, mg/L	0.20	0.07	0.06	0.01	0.02	0.29	**0.25**	**0.12**	**0.009**
F-calprotectin, mg/kg (n = 52)	0.00	0.02	0.30	0.00	0.07	0.06	**0.09**	**0.09**	**0.036**
SF-36bp scores^a^	**0.54**	**0.28**	**< 0.001**	**0.04**	**0.33**	**< 0.001**	**0.50**	**0.29**	**< 0.001**
HADS-D (n = 55)	**3.26**	**0.19**	**< 0.001**	**0.29**	**0.30**	**< 0.001**	**3.57**	**0.29**	**< 0.001**
IL-1RA, pg/mL	0.02	0.03	0.17	0.00	0.01	0.62	**0.03**	**0.14**	**0.005**
sIL-1RII, ng/mL	0.00	0.02	0.37	0.00	0.00	0.91	0.00	0.00	0.77
HSP90α, ng/mL (n = 52)	**0.73**	**0.10**	**0.03**	0.02	0.01	0.45	0.45	0.05	0.13
HMGB1, ng/mL (n = 52)	8.84	0.06	0.08	**0.84**	**0.11**	**0.02**	5.24	0.03	0.24
Anti-frHMGB1 abs^b^ (n = 49)	− **41.66**	**0.20**	**0.001**	− **2.43**	**0.14**	**0.01**	− **26.02**	**0.10**	**0.03**
PEDF, ng/mL (n = 46)	0.01	0.00	0.73	− 0.00	0.01	0.55	0.04	0.06	0.12
HPX, ng/mL (n = 46)	0.02	0.02	0.36	0.00	0.04	0.18	**0.03**	**0.11**	**0.027**

Univariable linear regression with fVAS, FSS, and SF-36vs, respectively, as dependent variables. Significant results in bold

^a^SF-36vs- and SF-36bp scores are reported as inverted values, with high numbers indicating low vitality/high bodily pain and low numbers indicating high vitality/low bodily pain. ^b^Absorbance at 490 nm

*abs* antibodies, *f-calprotectin* fecal calprotectin, *frHMGB1* fully reduced HMGB1, *fVAS* fatigue visual analogue scale, *FSS* Fatigue Severity Scale, *HADS-D* depression subscale of the Hospital Anxiety and Depression Scale, *HBI* Harvey Bradshaw Index, *HMGB1* high mobility group box 1, *HPX* hemopexin, *HSP* heat shock protein, *IL-1RA* interleukin-1 receptor antagonist, *sIL-1RII* soluble interleukin-1 receptor type 2, *PEDF* pigment epithelium-derived factor, *SES-CD* Simple Endoscopic Score for Crohn’s Disease, *SF-36* Medical Outcomes Study Short-form Health Survey, *SF-36bp* the bodily pain subscale of SF-36, *SF-36vs* the vitality subscale of SF-36

After multivariable regression analysis with forward and backward stepwise selection, HADS-D and SF-36bp scores remained as significant contributors to fatigue across all three fatigue instruments (Table 3). In addition, HSP90α contributed significantly in the fVAS model, HMGB1 in the FSS model, and IL-1RA in the SF-36vs model. Data from the full multivariable regression models are given in Additional file 1: Table S1.

**Table 3:** Three multivariable regression models^#^ with fVAS, FSS, and SF-36vs scores respectively as dependent variables

	fVAS			FSS			SF-36vs^a^	
	B	p-value		B	p-value		B	p-value
HADS-D	2.26	0.01	HADS-D	0.23	< 0.001	HADS-D	2.89	< 0.001
SF-36bp score^a^	0.48	< 0.001	SF-36bp score^a^	0.03	< 0.001	SF-36bp score^a^	0.38	< 0.001
HSP90α, ng/mL	0.72	0.01	HMGB1, ng/mL	0.56	0.047	IL-1RA, pg/mL	0.03	0.002
Model summary	R^2^ = 0.47, p < 0.001		Model summary	R^2^ = 0.56, p < 0.001		Model summary	R^2^ = 0.58, p < 0.001	

^#^Forward and backward selection yielded identical results. ^a^SF-36vs- and SF-36bp scores are reported as inverted values, with high numbers indicating low vitality/high bodily pain and low numbers indicating high vitality/low bodily pain. Data are from 56 patients with Crohn’s disease

*fVAS* fatigue visual analog scale, *FSS* Fatigue Severity Scale, *HADS-D* depression subscale of the Hospital Anxiety and Depression Scale, *HMGB1* high mobility group box 1, *HSP* heat shock protein, *IL-1RA* interleukin-1 receptor antagonist, *SF-36* Medical Outcomes Study Short-form Health Survey, *SF-36bp* the bodily pain subscale of SF-36, *SF-36vs* the vitality subscale of SF-36

**Fig. 1:**
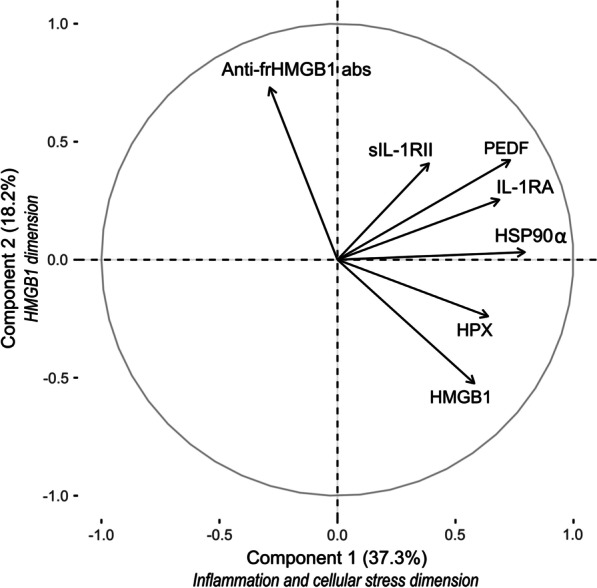
Unsupervised principal component analysis (PCA) of select potential fatigue-related biomolecules in plasma from 52 patients with Crohn’s disease. Component 1 is the “inflammation and cellular stress dimension” and Component 2 is the “HMGB1 dimension”. *abs* antibodies, *frHMGB1* fully reduced HMGB1, *HMGB1* high mobility group box 1, *HPX* hemopexin, *HSP* heat shock protein, *IL-1RA* interleukin-1 receptor antagonist, *sIL-1RII* soluble interleukin-1 receptor type 2, *PEDF* pigment epithelium-derived factor

To obtain a better understanding of the complex signaling network of biomolecules that influence fatigue, an unsupervised PCA was performed. The values were centered and scaled before analysis. Fifteen of the participants had one or more missing values (7.8% of the total values) that were imputed to enable the PCA. After Hotelling’s T-test for outliers, data from four patients were removed from the calculations. Two components with eigenvalues > 1 explained 55.5% of the variation in the dataset (Fig. 1, Additional file 2: Table S2). The first component explained 37.3% of the variation mainly contributed by IL-1RA, HSP90α, PEDF, and HPX (Additional file 3: Table S3). This component can be denoted as the “inflammation and cellular stress dimension”. The second component explaining 18.2% of the variation in the data set is dominated by HMGB1 and anti-frHMGB1 abs, the “HMGB1 dimension”.

**Fig. 2:**
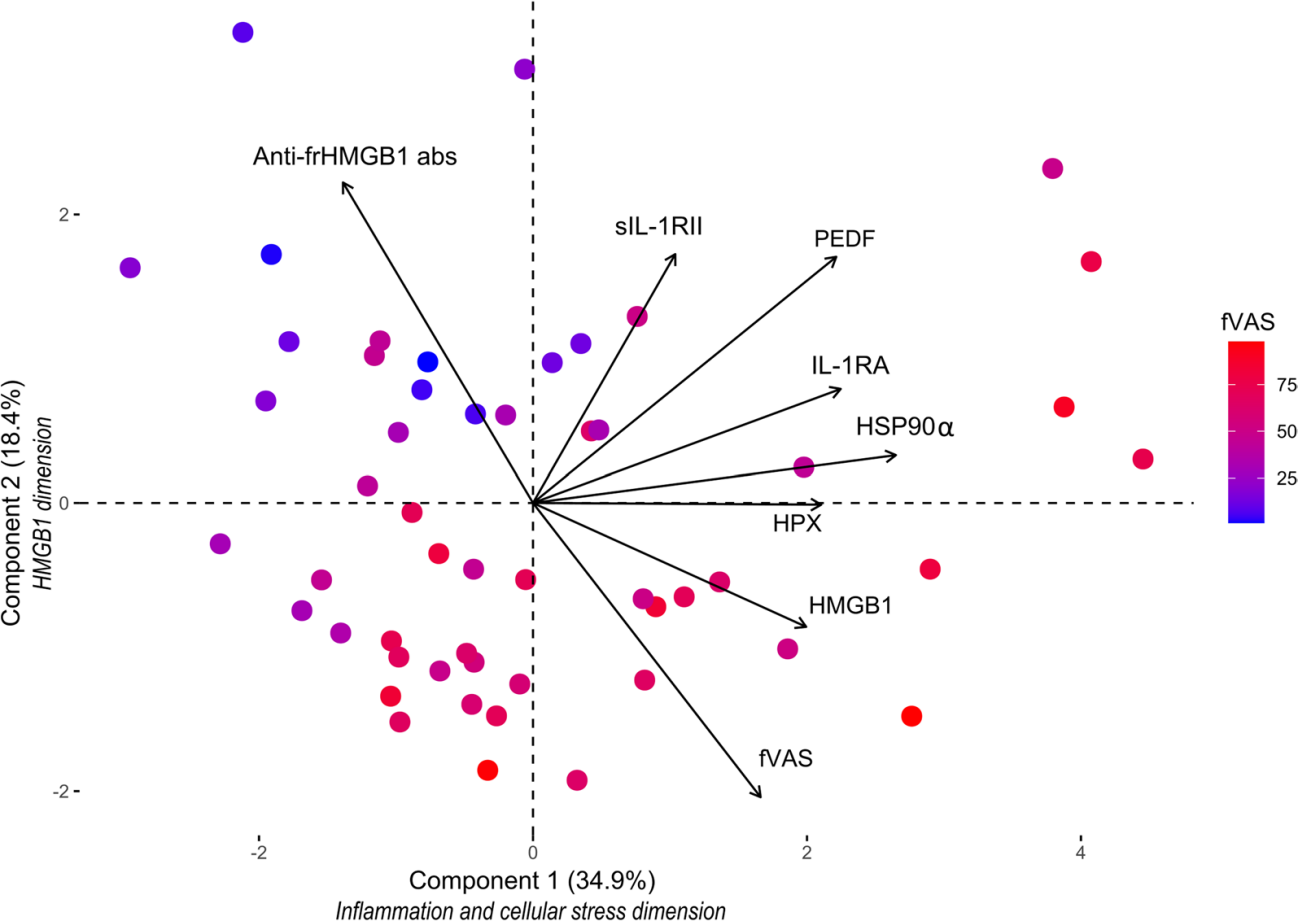
Model including fVAS in an unsupervised principal component analysis of 52 patients with Crohn’s disease showing the orientation and contribution of selected biomolecules measured in plasma. The individual points are colored according to the individuals’ fVAS scores. Component 1 is the “inflammation and cellular stress dimension” and Component 2 is the “HMGB1 dimension”. *abs* antibodies, *frHMGB1* fully reduced HMGB1, *fVAS* fatigue visual analogue scale, *HMGB1* high mobility group box 1, *HPX* hemopexin, *HSP* heat shock protein, *IL-1RA* interleukin-1 receptor antagonist, *sIL-1RII* soluble interleukin-1 receptor type 2, *PEDF* pigment epithelium-derived factor

The fVAS instrument has a wide response range (Wolfe 2004; Hewlett et al. 2011), therefore we selected scores from this instrument as an additional variable in a new unsupervised PCA model. This did not alter the results or orientation of the biplot substantially. In this plot, fVAS scores were predominantly associated with the HMGB1 dimension, and to a lesser extent the other biomolecules (Fig. 2, Additional files 4 and 5: Tables S4 and S5). Replacing fVAS scores with FSS or SF36vs scores did not alter the results in any meaningful way (data not shown).

## Discussion

The results of this study demonstrate how fatigue in patients with Crohn’s disease is influenced by a number of different psycho-emotional and biomolecular factors. The well-known effect of depressive mood and chronic pain is confirmed, the role of IL-1-related activity including HMGB1 is recognized, and we reveal that cellular stress responses that dampen inflammation and protect against cellular damage and death seem to be involved. We also confirmed that disease activity does not influence fatigue by applying objective disease measures, such as SES-CD, and classical inflammatory markers, such as f-calprotectin and CRP, using multivariable statistical models. This is in line with previous observations in both Crohn’s disease and other inflammatory diseases in which generic and unidimensional fatigue instruments have been applied (Pollard et al. 2006; Skoie et al. 2017; Azizoddin et al. 2019; Pope 2020; Grimstad et al. 2022; Skjellerudsveen et al. 2022; Hammer et al. 2022).

### Depression and pain

Depressed mood and pain made significant contributions to the severity of fatigue. This is a well-known effect observed across most studies of fatigue in which these two elements have been evaluated (Pollard et al. 2006; Miglianico et al. 2022). There are several possible explanations for why depression is linked to fatigue. First, fatigue and depression instruments share several claims and/or questions. Next, fatigue and depression share some dimensions in the concept of sickness behavior, and there are indications that depression can be induced through the same inflammatory mechanisms that induce fatigue; increased concentrations of IL-1β are found in both the brains of mice with mild depression and the blood of humans with depression disorders (Goshen et al. 2008; Ellul et al. 2016). Furthermore, pain is a strong factor for fatigue. According to the biological model of fatigue in which sickness behavior is the overarching concept, pain can be considered a warning sign of ongoing or impending tissue damage analogous to how pathogens can induce sickness behavior during infections (Inoue and Tsuda 2018; Omdal 2020).

### IL-1-related activity

Although the pathogenesis of fatigue is not fully understood, numerous studies have demonstrated the importance of IL-1β and IL-1-related molecules (Dantzer et al. 2008; Cavelti-Weder et al. 2011; Herlofson et al. 2018). IL-1β is released from innate immune cells, such as macrophages and microglia, upon activation by pathogens or other signals of immunological danger. IL-1β in the blood crosses the blood–brain barrier and binds to a complex of IL-1 receptor type 1 (IL-1RI) and a brain subtype of accessory protein (AcPb) (Smith et al. 2009). This is followed by neuronal activation and the generation of sickness behavior/fatigue. IL-1β levels in CSF reflect fatigue severity (Harboe et al. 2009; Lampa et al. 2012), but, due to very low concentrations in both the CSF and blood, two biomolecules that modulate IL-1 activity, IL-1RA and sIL-1RII, have been used as surrogates and more robust markers for IL-1β (Deuren et al. 1997; Yokoyama et al. 1998). IL-1RA binds to the IL-1 receptor on the cell surface and inhibits IL-1β from binding at the same site, whereas sIL-1RII is a soluble decoy receptor (Dinarello 2011). In the present study, IL-1RA made a small but significant contribution to fatigue in the final multivariable models only in the SF-36vs scores. However, in the PCA, both IL-1RA and sIL-1RII appeared to be driving forces for fatigue and indicate a logical but complex interaction of molecules.

### HMGB1

HMGB1 is a multifunctional protein. In its inactive form, it is a non-histone chromatin-binding protein present in all cell nuclei. It is passively released from necrotic or apoptotic cells following cellular stress and injury, or actively secreted by innate immune cells, such as macrophages and monocytes, after interactions with pathogens and endogenous DAMPs (Andersson et al. 2018; Gardella et al. 2002). Extracellular HMGB1 has the ability to function as an autoantigen and induce the production of autoantibodies against the protein in both autoimmune diseases and healthy subjects (Takaishi et al. 2012; Geng et al. 2020). This suggests a conserved immune-regulating mechanism that downregulates HMGB1-driven immune activation and inflammation. We have previously shown that high levels of anti-HMGB1 abs in patients with Crohn’s disease are associated with less fatigue (Kvivik et al. 2021). Taken together, the results suggest that HMGB1 is a driver of fatigue by itself and/or by enhancing IL-1 signaling, and that anti-HMGB1 abs reduce fatigue through the dampening of HMGB1-induced immune activation.

### Cellular stress proteins

Heat shock proteins are evolutionarily well conserved chaperone proteins with cell-protective functions. During infections, highly reactive oxygen species are produced to kill pathogens in the phagolysosomes; thus, strong protective mechanisms are necessary to maintain the cell and homeostasis. HSPs play central roles in the downregulation of inflammation. HSP90α, the inducible isoform of this protein, is released in response to a variety of cellular stressors, including inflammation and thermal stress, and downregulates innate immune responses (Calderwood et al. 2016). HSP90α is associated with fatigue severity in both patients with primary Sjögren’s syndrome and patients with Crohn’s disease, and higher plasma concentrations of HSP90α are seen in patients with more severe fatigue (Grimstad et al. 2020; Bårdsen et al. 2016). A hypothetical mechanism is that HSP90α crosses the blood–brain barrier and binds to TLR4 on microglia (Hines et al. 2013). The microglia become activated and release IL-1β, which can bind to nearby neuronal IL-1R1/AcPb complexes and induce sickness behavior (Smith et al. 2009).

PEDF (encoded by *SERPINF1*) is a secreted glycoprotein belonging to the large serpin family. These proteins were originally named for their function as serine proteinase inhibitors, but several of the large serpin family members are not inhibitors, but rather chaperones, and may have other protective features. PEDF is a multifunctional protein and has anti-angiogenic, anti-tumorigenic, and neurotrophic functions (Tombran-Tink and Barnstable 2003). The protein plays an important role in mediating cellular protection during exposure to oxidative stress and inflammation by preventing stress-induced angiogenesis and apoptosis (Brook et al. 2019; Sanchez et al. 2012). Both animal and human studies have found that increased concentrations of PEDF in CSF and plasma are associated with major depression (Ditzen et al. 2012; Ryan et al. 2017; Tian et al. 2020).

On the other hand, studies have shown that PEDF may promote inflammation in rat microglial cells via the activation of NF-κB or cAMP-responsive element binding protein (CREB) signaling and up-regulating of pro-inflammatory genes, including IL-1β, IL-6, and TNF-α (Sanagi et al. 2005). Similarly, another study found that PEDF activates NF-κB, CREB, and activator protein 1 (AP-1) signaling, inducing pro-inflammatory gene expression of IL-1β, IL-6, TNF-α, macrophage inflammatory protein (MIP)-α, and MIP-3α in cultured neonatal rat astrocytes (Yabe et al. 2005). This shows that PEDF is a multifunctional protein with different functions depending on the site and tissues involved (Brook et al. 2019). Therefore, increased production of pro-inflammatory cytokines in brain tissue could potentially explain the association with fatigue observed in the PCA (Fig. 2).

HPX is an acute phase protein with high affinity for heme. Its main function is transportation of heme from the plasma to the liver. Heme has the potential to intercalate into lipid membranes and produce highly toxic hydroxyl radicals, which HPX counteracts. HPX also protects cells from oxidative stress during inflammatory conditions (Tolosano and Altruda 2002). Higher levels of HPX have been associated with depression (Frye et al. 2015).

A previous explorative proteomic study in patients with primary Sjögren’s syndrome associated CSF HPX and PEDF concentrations with fatigue (Larssen et al. 2019). In this study, 15 proteins with functions mostly related to innate immunity and cellular defense responses were able to separate groups of patients with severe vs. mild fatigue. In addition, both HPX and PEDF were among 20 proteins in the CSF that were associated with Persian Gulf War Illness in soldiers, an equivalent to chronic fatigue syndrome. PEDF was one of five proteins that predicted chronic fatigue syndrome with > 80% concordance (Baraniuk et al. 2005).

### Limitations

Because sickness behavior and fatigue are generated in the brain, CSF would have been a more optimal matrix to investigate candidate biomolecules. However, the procedure for obtaining CSF is invasive, and access to CSF from patients is limited. In addition, it is possible that the concentrations of the selected biomolecules may vary over time during the disease course. This could have varying impact on fatigue severity, which could not be evaluated in this study. As for the verification of the PCA, our findings should be validated in independent studies. This study’s strength is that the participants constitute a well-defined, newly diagnosed patient group not influenced by various drug regimens.

## Conclusion

In conclusion, our study highlights the complexity of biomolecules that influence fatigue. In addition to the well-known modulation by mental depression and pain, HMGB1 and other pro-and anti-inflammatory proteins participate in a network that contribute to the generation and regulation of fatigue.

## Supplementary Information

**Additional file 1: Table S1.** Full multivariable regression models with fVAS, FSS, and SF-36vs scores respectively as dependent variables.

**Additional file 2: Table S2.** Amount of variation explained by each component in an unsupervised principal component analysis of 52 patients with Crohn’s disease.

**Additional file 3: Table S3.** Association (coordinates) and contribution of all variables to the first and second component in the unsupervised principal component analysis of 52 patients with Crohn’s disease.

**Additional file 4: Table S4.** Amount of variation explained by each component in the model including fVAS in an unsupervised principal component analysis of 52 patients with Crohn’s disease.

**Additional file 5: Table S5.** Association (coordinates) and contribution of all variables to the first and second component in the model including fVAS in an unsupervised principal component analysis of 52 patients with Crohn’s disease.

## Data Availability

The datasets used and analyzed during the current study are available from the corresponding author on reasonable request.

## Abbreviations

abs: Antibodies
AcPb: IL-1RI accessory protein in the brain
AP-1: Activator protein 1
CREB: CAMP-responsive element binding protein
CRP: C-reactive protein
CSF: Cerebrospinal fluid
CV: Coefficient of variation
DAMP: Damage-associated molecular pattern
dsHMGB1: Disulfide HMGB1
f-calprotectin: Fecal calprotectin
frHMGB1: Fully reduced HMGB1
fVAS: Fatigue visual analog scale
FSS: Fatigue Severity Scale
HADS-D: Depression subscale of the Hospital Anxiety and Depression Scale
HBI: Harvey Bradshaw Index
HMGB1: High mobility group box 1
HPX: Hemopexin
HSP90α: Heat shock protein 90 alpha
IL: Interleukin
IL-1RI: Interleukin-1 receptor type 1
IL-1RA: Interleukin-1 receptor antagonist
sIL-1RII: Soluble interleukin-1 receptor type 2
LOD: Limit of detection
MD-2: Myeloid differentiation factor 2
MIP: Macrophage inflammatory protein
PCA: Principal component analysis
PEDF: Pigment epithelium-derived factor
RAGE: Receptor for advanced glycation end products
SD: Standard deviation
SES-CD: Simple Endoscopic Score for Crohn’s Disease
SF-36: Medical Outcomes Study Short-form Health Survey
SF-36bp: The bodily pain subscale of SF-36
SF-36vs: The vitality subscale of SF-36
TLR4: Toll-like receptor 4
TNF: Tumor necrosis factor
