# Supplementary material for: High mobility group box 1 and a network of other biomolecules influence fatigue in patients with Crohn’s disease
Source: Mol Med. 2023 Jun 26;29:81. doi: 10.1186/s10020-023-00679-6 (PMC10291761; doi:10.1186/s10020-023-00679-6)
Supplement: Supplementary file 1 — Additional file 1: Table S1. Full multivariable regression models with fVAS, FSS, and SF-36vs scores respectively as dependent variables. [file 10020_2023_679_MOESM1_ESM.docx]

Table S1. Full multivariable regression models with fVAS, FSS, and SF-36vs scores respectively as dependent variables.

|  | **fVAS** | |  | **FSS** | |  | **SF-36vs^a^** | |
| --- | --- | --- | --- | --- | --- | --- | --- | --- |
|  | B | p-value |  | B | p-value |  | B | p-value |
| HBI | −0.47 | 0.62 | HBI | 0.01 | 0.84 | HBI | -0.20 | 0.81 |
| CRP, mg/L | 0.03 | 0.76 | SES-CD | 0.00 | 0.99 | SES-CD | 0.50 | 0.26 |
| HADS-D | 2.42 | **0.015** | F-calprotectin, mg/kg | 0.00 | 0.69 | CRP, mg/L | -0.04 | 0.78 |
| SF-36bp, score^a^ | 0.40 | **0.007** | HADS-D | 0.22 | **0.002** | F-calprotectin, mg/kg | 0.00 | 0.86 |
| HSP90α, ng/mL | 0.65 | 0.06 | SF-36bp score^a^ | 0.03 | **0.012** | HADS-D | 2.78 | **0.001** |
| HMGB1, ng/mL | -0.57 | 0.90 | HMGB1, ng/mL | 0.45 | 0.18 | SF-36bp score^a^ | 0.36 | **0.006** |
| Anti-frHMGB1 abs^b^ | -19.67 | 0.12 | Anti-frHMGB1 abs^b^ | -0.65 | 0.47 | IL-1RA, pg/mL | 0.02 | 0.09 |
|  |  |  | HPX, ng/mL | 0.00 | 0.92 | Anti-frHMGB1 abs^b^ | -2.64 | 0.81 |
|  |  |  |  |  |  | HPX, ng/mL | 0.01 | 0.47 |
| **Model summary** | **R^2^ = 0.51, p < 0.001** | | **Model summary** | **R^2^ = 0.57, p < 0.001** | | **Model summary** | **R^2^ = 0.62, p < 0.001** | |

^a^SF-36vs and SF-36bp scores are reported as inverted values, with high numbers indicating low vitality/high bodily pain and low numbers indicating high vitality/low bodily pain. ^b^Absorbance at 490 nm. Significant results in bold. Data are from 56 patients with Crohn’s disease.

abs: antibodies; f-calprotectin: fecal calprotectin; frHMGB1: fully reduced HMGB1; fVAS: fatigue visual analogue scale; FSS: Fatigue Severity Scale; HADS-D: depression subscale of the Hospital Anxiety and Depression Scale; HBI: Harvey Bradshaw Index; HMGB1: high mobility group box 1; HPX: hemopexin; HSP: heat shock protein; IL-1RA: interleukin-1 receptor antagonist; SES-CD: Simple Endoscopic Score for Crohn’s Disease; SF-36: Medical Outcomes Study Short-form Health Survey; SF-36bp: the bodily pain subscale of SF-36; SF-36vs: the vitality subscale of SF-36.
