# Supplementary material for: High mobility group box 1 and a network of other biomolecules influence fatigue in patients with Crohn’s disease
Source: Mol Med. 2023 Jun 26;29:81. doi: 10.1186/s10020-023-00679-6 (PMC10291761; doi:10.1186/s10020-023-00679-6)
Supplement: Supplementary file 2 — Additional file 2: Table S2. Amount of variation explained by each component in an unsupervised principal component analysis of 52 patients with Crohn’s disease. [file 10020_2023_679_MOESM2_ESM.docx]

Table S2. Amount of variation explained by each component in an unsupervised principal component analysis of 52 patients with Crohn’s disease.

| Component | 1 | 2 | 3 | 4 | 5 | 6 | 7 |
| --- | --- | --- | --- | --- | --- | --- | --- |
| Variance | 2.61 | 1.28 | 0.91 | 0.79 | 0.66 | 0.39 | 0.37 |
| % of variance | 37.3 | 18.2 | 13.0 | 11.3 | 9.4 | 5.6 | 5.2 |
| Cumulative % of variance | 37.3 | 55.5 | 68.5 | 79.8 | 89.2 | 94.8 | 100 |
