# Supplementary material for: High mobility group box 1 and a network of other biomolecules influence fatigue in patients with Crohn’s disease
Source: Mol Med. 2023 Jun 26;29:81. doi: 10.1186/s10020-023-00679-6 (PMC10291761; doi:10.1186/s10020-023-00679-6)
Supplement: Supplementary file 3 — Additional file 3: Table S3. Association (coordinates) and contribution of all variables to the first and second component in the unsupervised principal component analysis of 52 patients with Crohn’s disease. [file 10020_2023_679_MOESM3_ESM.docx]

Table S3. Association (coordinates) and contribution of all variables to the first and second components in the unsupervised principal component analysis of 52 patients with Crohn’s disease.

| Component | 1 | | 2 | |
| --- | --- | --- | --- | --- |
|  | Coordinates | Contribution | Coordinates | Contribution |
| IL-1RA | 0.69 | 18.01 | 0.25 | 5.07 |
| sIL-1RII | 0.39 | 5.75 | 0.41 | 13.05 |
| HSP90α | 0.79 | 24.13 | 0.03 | 0.08 |
| HMGB1 | 0.58 | 12.89 | -0.52 | 21.53 |
| Anti**-**frHMGB1 abs | -0.29 | 3.15 | 0.73 | 41.77 |
| PEDF | 0.73 | 20.48 | 0.42 | 13.98 |
| HPX | 0.64 | 15.59 | -0.24 | 4.52 |

abs: antibodies; frHMGB1: fully reduced HMGB1; HMGB1: high mobility group box 1; HPX: hemopexin; HSP: heat shock protein; IL-1RA: interleukin-1 receptor antagonist; sIL-1RII: soluble interleukin-1 soluble receptor type 2; PEDF: pigment epithelium-derived factor.
