# Supplementary material for: High mobility group box 1 and a network of other biomolecules influence fatigue in patients with Crohn’s disease
Source: Mol Med. 2023 Jun 26;29:81. doi: 10.1186/s10020-023-00679-6 (PMC10291761; doi:10.1186/s10020-023-00679-6)
Supplement: Supplementary file 4 — Additional file 4: Table S4. Amount of variation explained by each component in the model including fVAS in an unsupervised principal component analysis of 52 patients with Crohn’s disease. [file 10020_2023_679_MOESM4_ESM.docx]

Table S4. Amount of variation explained by each component in the model including fVAS in an unsupervised principal component analysis of 52 patients with Crohn’s disease.

| Component | 1 | 2 | 3 | 4 | 5 | 6 | 7 | 8 |
| --- | --- | --- | --- | --- | --- | --- | --- | --- |
| Variance | 2.79 | 1.48 | 0.91 | 0.89 | 0.72 | 0.47 | 0.39 | 0.36 |
| % of variance | 34.9 | 18.4 | 11.4 | 11.1 | 9.0 | 5.8 | 4.9 | 4.5 |
| Cumulative % of variance | 34.9 | 53.3 | 64.7 | 75.8 | 84.8 | 90.6 | 95.5 | 100 |
