# Supplementary material for: High mobility group box 1 and a network of other biomolecules influence fatigue in patients with Crohn’s disease
Source: Mol Med. 2023 Jun 26;29:81. doi: 10.1186/s10020-023-00679-6 (PMC10291761; doi:10.1186/s10020-023-00679-6)
Supplement: Supplementary file 5 — Additional file 5: Table S5. Association (coordinates) and contribution of all variables to the first and second component in the model including fVAS in an unsupervised principal component analysis of 52 patients with Crohn’s disease. [file 10020_2023_679_MOESM5_ESM.docx]

Table S5. Association (coordinates) and contribution of all variables to the first and second component in the model including fVAS in an unsupervised principal component analysis of 52 patients with Crohn’s disease.

| Component | 1 | | 2 | |
| --- | --- | --- | --- | --- |
|  | Coordinates | Contribution | Coordinates | Contribution |
| fVAS | 0.50 | 8.87 | -0.61 | 25.33 |
| IL-1RA | 0.67 | 16.21 | 0.24 | 3.78 |
| sIL-1RII | 0.31 | 3.46 | 0.52 | 18.07 |
| HSP90α | 0.79 | 22.54 | 0.10 | 0.66 |
| HMGB1 | 0.60 | 12.77 | -0.26 | 4.49 |
| Anti-frHMGB1 abs | -0.42 | 6.18 | 0.67 | 30.00 |
| PEDF | 0.66 | 15.74 | 0.51 | 17.68 |
| HPX | 0.63 | 14.22 | -0.00 | 0.00 |

abs: antibodies; frHMGB1: fully reduced HMGB1; fVAS: fatigue visual analogue scale; HMGB1: high mobility group box 1; HPX: hemopexin; HSP: heat shock protein; IL-1RA: interleukin-1 receptor antagonist; sIL-1RII: soluble interleukin-1 receptor type 2; PEDF: pigment epithelium-derived factor.
